# Models for successful interactions of psychiatrists with indigenous patients and communities

**DOI:** 10.1192/j.eurpsy.2021.861

**Published:** 2021-08-13

**Authors:** L. Mehl-Madrona, B. Mainguy

**Affiliations:** 1 Medical Arts And Humanities Program, University of Maine, Orono, United States of America; 2 Education Division, Coyote Institute - Canada, Ottawa, Canada

**Keywords:** Participatory action research, OCAP Principles, two-eyed seeing, Indigenous communities

## Abstract

**Introduction:**

Conventional psychiatric services are not always acceptable to indigenous communities and people.

**Objectives:**

To present successful models of interactions of psychiatrists with indigenous patients and communities based upon our work with five communities in Maine.

**Methods:**

We reviewed the strategies that worked for community interaction from our project for supporting indigenous communities to implement medication-assisted treatment and we reviewed the literature to see what other strategies are reported successful.

**Results:**

Psychiatrists working in these communities may need to share more personal details than to what they are usually accustomed to be accepted. They may need to acknowledge local culture and spirituality and work with traditional knowledge holders to create collaborative healing approaches. As part of this, a narrative approach appeared to work best in which the psychiatrist worked within the stories and beliefs of the community which required taking the time in dialogue to learn those stories and beliefs. Specifically, we address the challenges of flying into northern, rural, and remote communities, of academic physicians consulting to tribal-based opiate treatment programs, of modifying usual counseling techniques such as motivational interviewing to an indigenous population, and of the changes made in practice styles when taking into account the critiques made by indigenous people about medicine in general and psychiatry in particular.

**Conclusions:**

We propose that participatory action-based approaches can improve service delivery to indigenous people. Indigenous cultures share a collectivist mindset in which the needs of the group supersede the needs of individuals, a reliance upon stories, and commitment to a biopsychosocial and spiritual approach.

